# Associations of very low Lipoprotein(a) levels with risks of new-onset diabetes and non-alcoholic liver disease

**DOI:** 10.1016/j.athplu.2024.07.001

**Published:** 2024-07-11

**Authors:** Ming Wai Yeung, M. Abdullah Said, Yordi J. van de Vegte, Niek Verweij, Robin P.F. Dullaart, Pim van der Harst

**Affiliations:** a Department of Cardiology University of Groningen, University Medical Center Groningen, 9700 RB Groningen, the Netherlands; b Department of Cardiology, Division of Heart & Lungs, University Medical Center Utrecht, University of Utrecht, Utrecht, the Netherlands; c Department of Endocrinology, University of Groningen, University Medical Center Groningen, Groningen, the Netherlands

**Keywords:** Cardiovascular risk factor, Lipoprotein(a), Type 2 diabetes, NAFLD, Mendelian randomization, Prospective study, UK biobank

## Abstract

**Background and aims:**

We aimed to study the association of very low serum Lipoprotein(a) [Lp(a)] concentrations with new-onset type 2 diabetes (T2D) and non-alcoholic liver disease (NAFLD) in the context of statin usage in the UK Biobank, a large prospective population cohort.

**Methods:**

Using an extended biomarker dataset, we identified 47,362 participants with very low Lp(a) concentrations (<3.8 nmol/L) from a total of 451,479 participants. With a median follow-up of 12.3 years, we assessed the risk of new-onset cardiometabolic diseases in participants stratified by statin usage with Cox proportional hazards models. We performed two-sample Mendelian randomization MR analyses to test causal relationship between genetically predicted Lp(a) and T2D and NAFLD.

**Results:**

Taking the participants with Lp(a) within reportable range as the reference group, the hazard ratios (HR) for T2D were 1.07 (95 % confidence interval, CI 1.01–1.13) and for NAFLD 1.30 (95 % CI 1.20–1.41) respectively for participants with very low Lp(a) (<3.8 nmol/L). The risk for new-onset T2D was higher in participants using statins (adjusted HR 1.15; 95 % CI 1.05–1.27). The risk estimates for new-onset NAFLD were comparable in the analysis stratified by statin use. There was no evidence for causal links between genetically predicted Lp(a) and T2D nor NAFLD in two-sample MR analyses.

**Conclusions:**

Very low Lp(a) was associated with higher risks of T2D and NAFLD in a prospective analysis of the UK Biobank. The association with T2D was influenced by lipid lowering medication usage. MR analyses did not support causality for these inverse associations.

## Graphical abstract

**Figure:**
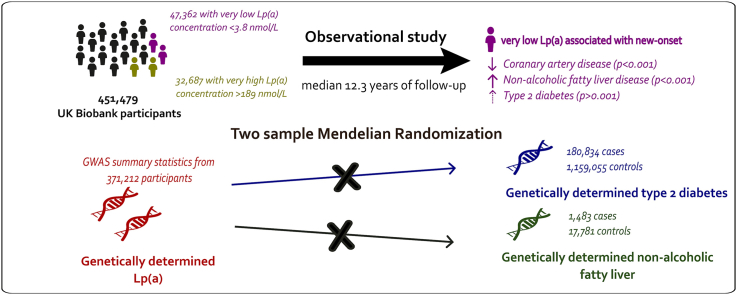

## 1 Introduction

Lipoprotein(a) [Lp(a)] is a specific lipoprotein produced in the liver that consists of a polymorphic glycoprotein, called apolipoprotein(a) [apo(a)] linked to apolipoprotein-B(100) on an LDL particle in a 1:1 ratio. Ample evidence supports a causal association between elevated Lp(a) and various cardiovascular diseases (CVD) [[1], [2], [3]]. Interestingly, recent epidemiological studies raised the possibility that very low Lp(a) (<7.5–12.5 nmol/L) is associated with increased risk of new-onset type 2 diabetes mellitus (T2D) [1]. However, it is unclear whether Lp(a) is a mere marker or whether there is a causal mechanism behind this possible inverse relationship. An inverse association has also been suggested for fatty liver disease [4]. Closely linked to metabolic dysfunction, the causal relationship between non-alcoholic liver disease (NAFLD) (new nomenclature: metabolic associated steatotic fatty liver disease [MASLD]) and diabetes has been supported by evidence from both epidemiological studies and animal studies [[5], [6], [7]]. The opposed direction of the Lp(a) association with new-onset T2D, NAFLD and CVDs also raises the question whether the risk of diabetes might be modified by statin treatment [8].

In the current study, we aimed to investigate Lp(a) associations with new-onset T2D and NAFLD in the UK Biobank, a large population-based cohort, and examined effect modification by treatment with statin. We further examined the potential causal relationship of Lp(a) with T2D and NAFLD using a two-sample MR approach.

## 2 Methods

### 2.1 Study population

The UK Biobank study is a population-based prospective cohort in the United Kingdom in which over 500,000 participants aged between 40 and 69 years were included between 2006 and 2010. All participants have given informed consent for this study. The UK Biobank has ethical approval from North West - Haydock Research Ethics Committee (REC reference: 16/NW/0274). Details of the UK Biobank study has been described in detail previously [9]. This research has been conducted using the UK Biobank Resource under Application Number 74395. Demographics including age, sex, smoking status, alcohol consumption and ethnic background were collected at the baseline visit (Table S1).

### 2.2 Ascertainment of health outcomes

Ascertainment of diseases and medication usage in the UK Biobank was based on a combination of hospital inpatient records, primary care records and self-reported records during an interview with a trained staff (Table S2). Briefly, T2D diagnosis was captured using ICD10 code E10-E14 or equivalent diagnosis codes; NAFLD diagnosis was captured using K721, K740, K741, K742, K746, K758 and K760 or equivalent diagnosis codes [10]. Participants with prevalent disease at baseline per outcome were excluded from the regression analysis. For T2D, we additionally excluded participants with baseline serum glycated haemoglobin (HbA1c) ≥6.5 % (48 mmol/mol). Health outcome data were processed and extracted using the ukbpheno v1.0 package in R [11]. Participant follow-up started at inclusion and ended at date of event, death or last recorded follow-up (Table S3), whichever occurred first.

### 2.3 Serum biomarker measurement and extreme Lp(a) concentration

**Fig. 1:**
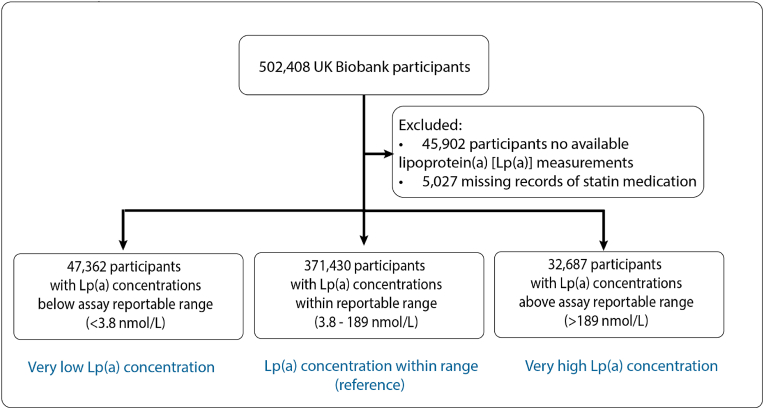
Study population.

While typical Lp(a) concentration ranges between 0.2 and 750 nmol/L in humans [1], the Lp(a) concentrations of the UK Biobank participants were measured using an immuno-turbidimetric assay (Randox Bioscience, UK) with a reportable range 3.8–189 nmol/L. Notably >80,000 participants had their values outside of reportable range, accounting for 17.5 % of participants with measured Lp(a) concentrations. While an accurate quantification of Lp(a) concentration could not be established, this information is invaluable for the identification of participants with extreme Lp(a) concentrations. We therefore obtained Lp(a) concentrations outside of the reportable range from the UK Biobank (Return 2321). We considered participants to have very low Lp(a) if they had Lp(a) concentrations <3.8 nmol/L; and those with Lp(a) concentrations >189 nmol/L very high Lp(a) (Fig. 1).

Non-fasting venous blood samples were drawn during participants' baseline visit to the Assessment Centre [12]. Low density lipoprotein cholesterol (LDL-C) was measured using an enzymatic selective protection method while high density lipoprotein cholesterol (HDL-C) was measured using an enzyme immuno-inhibition method. Triglycerides were measured using an enzymatic method, namely the glycerol-3-phosphate (GPO)-peroxidase (POD) chromogenic method. All biomarkers were measured on a Beckman Coulter AU5800 (Beckman Coulter [UK], Ltd). Glycated haemoglobin (HbA1c) was measured in mmol/mol with high performance liquid chromatography method (Bio-Rad Laboratories, Inc.) on Bio-Rad Variant II Turbo analysers [13].

### 2.4 Statistical analyses

Data are presented as median (IQR) for continuous variables and as counts with percentages for discrete and categorical variables, respectively.

#### 2.4.1 Cox regression analyses

We performed two Cox proportional hazards regression analyses to test the association of Lp(a) with coronary artery disease (CAD), as compared to T2D and NAFLD separately, stratified by statin use. We first examined the disease risk by Lp(a) categories. We estimated HRs with 95 % confidence intervals in participants with either very low or very high Lp(a) concentrations against participants with Lp(a) concentrations between 3.8 and 189 nmol/L. In the second analysis, we directly modelled the inverse rank normalized serum Lp(a) concentrations linearly only in participants with Lp(a) in the reported range. In both analyses, we considered five models. Model 1 was adjusted for age at baseline and sex; model 2 was further adjusted for body mass index (BMI) and hypertension; model 3 was further adjusted for LDL-C and HDL-C; model 4 for for triglycerides and finally model 5 was further adjusted for alcohol use. We additionally examined if these associations were affected by use of statin, a lipid-lowering medication with reported link to increased risk of diabetes [14]. Cox regressions were performed using Stata 17.1 (StataCorp LLC). We considered results with *p* < 0.001 to be statistically significant to correct for multiple testing.

#### 2.4.2 MR analyses

We performed two-sample Mendelian randomization (MR) analyses to assess potential causal effects of Lp(a) on T2D and NAFLD. We took the lead SNPs identified in the UK Biobank GWAS as instrumental variables for Lp(a) [3]. We took estimates from the summary statistics data of the DIAMANTE Consortium (1,159,055 controls and 180,834 [13.5 %] cases) for diabetes [15] and EPoS Consortium for NAFLD (17,781 controls and 1483 [7.7 %] histologically confirmed cases) [16]. SNPs (genetic instruments) that were not available in the outcome GWAS were replaced with proxies in linkage disequilibrium (LD) of r^2^>0.8 respectively or excluded from the MR if no eligible proxies were available. Harmonization of SNP effects and the MR analyses were performed using R (version 4.0.3), the TwoSampleMR package (version 0.5.6) [17], MendelianRandomization (version 0.6.0) [18], MRMix (version 0.1.0) [19], MR-PRESSO (Pleiotropy Residual Sum and Outlier) (version 1.0) [20] and mr.raps (version 0.2) [21].

MR estimates were obtained using an inverse variance weighted (IVW)-fixed effects model followed by sensitivity analyses to assess the robustness of the findings of the IVW estimates. For this, we used IVW-random effects, MR-Egger [22], median-, mode-based estimator MR analyses, MR-PRESSO, MR-Lasso [23], MR-Mix and MR-RAPS. Weak instrument bias was tested using F-statistics [24], with F-statistics >10 indicating low risk of weak instrument bias. SNPs with F-statistics ≤10 were excluded from the analyses. The I^2^ index (>25 %) [25] and Cochran's Q statistic (P < 0.05) [26] were determined to assess potential heterogeneity within IVW-fixed effects estimates, indicating at least balanced horizontal pleiotropy for some of the SNPs.

The MR-Egger method allows for a non-zero intercept hence capturing the unbalanced horizontal pleiotropic effects, if these pleiotropic effects are independent of their association with the exposure of interest (Instrument Strength Independent of Direct Effect [InSIDE] assumption) [27]. Rücker's Q'was calculated to assess heterogeneity within MR-Egger analyses, and the difference between Rücker's Q′ statistic and Cochran's Q statistic (Q-Q′) was tested [26]. The MR-Egger intercept was tested for a deviation from zero. We consider presence of unbalanced horizontal pleiotropy in the in the case of a significant Q-Q′ and a non-zero MR-Egger intercept. The MR-Egger estimate was considered the causal estimate rather than the MR-IVW method in presence of unbalanced horizontal pleiotropy if the general InSIDE assumption holds (Fig. S1).

We additionally assessed the potential weak instrument bias in the MR-Egger analyses by calculating the I^2^_GX_, which is the true variance of the SNP-exposure association and indicates low risk of measurement error if >95 % [27].

The median/mode-based methods take, respectively, the median or mode of the ratio estimates instead of the weighted mean in IVW method. They are therefore robust against the presence of a limited number of invalid instruments. MR-PRESSO method performs the IVW method after exclusion of SNPs with MR estimates that differ substantially from the rest, thereby adjusting for the potential pleiotropy [20]. Similarly, the MR-Lasso method is another augmented version of the IVW method with outliers removed. MR-RAPS and MR-Mix methods adjust the estimates to address the violation of valid instrument assumption.

## 3 Results

We studied 451,479 participants (54.3 % women with mean age 57.1 years) with Lp(a) measurements and information on statin usage. Among these participants, 371,430 had Lp(a) measurements within the reportable range of 3.8–189 nmol/L (median 21.1; IQR 9.6–61.9); 47,362 participants had very low Lp(a) concentrations (<3.8 nmol/L) while 32,687 participants had very high Lp(a) (>189 nmol/L). A total of 83,405 (18.5 %) participants were on statin at baseline. Participants with very low Lp(a) concentrations were more likely to be White men and had higher median weekly alcohol consumption, had lower LDL-C and HDL-C but higher triglycerides (Table 1).

**Table 1:** Clinical characteristics of UKB participants by Lp(a) concentration.

Variable	Lp(a) < 3.8 nmol/L	3.8≤ Lp(a) ≤189 nmol/L	Lp(a) > 189 nmol/L	*p*
N	47,362	371,430	32,687	
Age at baseline visit, years (IQR)	57.57 (49.69, 63.43)	58.24 (50.48, 63.71)	59.71 (52.77, 64.31)	<0.001
Sex				<0.001
Men	24734 (52.2 %)	168787 (45.4 %)	12612 (38.6 %)	
Women	22628 (47.8 %)	202643 (54.6 %)	20075 (61.4 %)	
Ethnicity				<0.001
White	46110 (97.4 %)	348537 (93.8 %)	30811 (94.3 %)	
Asian	545 (1.2 %)	9224 (2.5 %)	447 (1.4 %)	
Black	86 (0.2 %)	6094 (1.6 %)	879 (2.7 %)	
Mixed	167 (0.4 %)	2315 (0.6 %)	197 (0.6 %)	
Other/Unknown	454 (1.0 %)	5260 (1.4 %)	353 (1.1 %)	
Current smoker at baseline visit	5054 (10.7 %)	39389 (10.6 %)	3389 (10.4 %)	0.35
Alcohol consumption in UK Units (8g pure alcohol) per week at baseline visit	12.40 (4.80, 23.80)	11.20 (4.25, 21.90)	11.20 (4.20, 22.40)	<0.001
BMI at baseline visit, kg/m^2^	27.09 (24.30, 30.48)	26.69 (24.10, 29.81)	26.89 (24.38, 30.00)	<0.001
Systolic blood pressure at baseline visit, mmHg	133.03 (121.55, 145.47)	131.71 (120.21, 144.47)	133.05 (121.13, 145.50)	<0.001
Diastolic blood pressure at baseline visit, mmHg	82.13 (76.47, 88.19)	81.72 (76.06, 87.39)	81.73 (76.06, 87.79)	<0.001
LDL-C at baseline visit, mg/dl	128.83 (107.49, 151.45)	135.75 (113.91, 158.68)	145.30 (120.37, 170.71)	<0.001
HDL-C at baseline visit, mg/dl	52.74 (43.85, 63.64)	54.02 (45.28, 64.65)	56.30 (47.37, 66.93)	<0.001
Triglycerides at baseline visit, mg/dl	136.67 (94.33, 201.33)	130.82 (92.29, 189.37)	127.19 (91.59, 180.87)	<0.001
Hypertension at baseline visit	15112 (32.0 %)	113374 (30.6 %)	12168 (37.3 %)	<0.001
Statin usage at baseline visit	8477 (17.9 %)	65177 (17.5 %)	9751 (29.8 %)	<0.001
New-onset T2D	5189 (11.0 %)	32079 (8.7 %)	3076 (9.4 %)	<0.001
New-onset non-alcoholic fatty liver disease	1102 (2.3 %)	5586 (1.5 %)	429 (1.3 %)	<0.001
New-onset coronary artery disease	4449 (9.4 %)	36438 (9.8 %)	5005 (15.3 %)	<0.001
New-onset aortic stenosis	424 (0.9 %)	3662 (1.0 %)	641 (2.0 %)	<0.001
New-onset heart failure	1603 (3.4 %)	11984 (3.2 %)	1404 (4.3 %)	<0.001
New-onset stroke	1993 (4.2 %)	15627 (4.2 %)	1719 (5.3 %)	<0.001

BMI: body mass index, HDL-C: high density lipoprotein cholesterol, IQR: interquartile range, LDL-C: low-density lipoprotein cholesterol.

During a median follow-up of 12.3 years (IQR 11.5–13.1) 40,344 participants were diagnosed with T2D and 7,117 with NAFLD. There were 45,892 cases of new-onset CAD. There was more new-onset diabetes in participants with the lowest Lp(*a*) namely 11.0% in participants with Lp(a) concentrations <3.8 nmol/L, 8.7% in participants with Lp(a) concentrations between 3.8 and 189 nmol/L and 9.4% in participants with Lp(a) > 189 nmol/L respectively. We observed more new-onset NAFLD in participants with Lp(a) concentrations <3.8 nmol/L (2.3 %), in contrast to 1.5% among participants with Lp(a) concentrations between 3.8 and 189 nmol/L and 1.3% among participants with Lp(a) > 189 nmol/L respectively (Table 1).

**Fig. 2:**
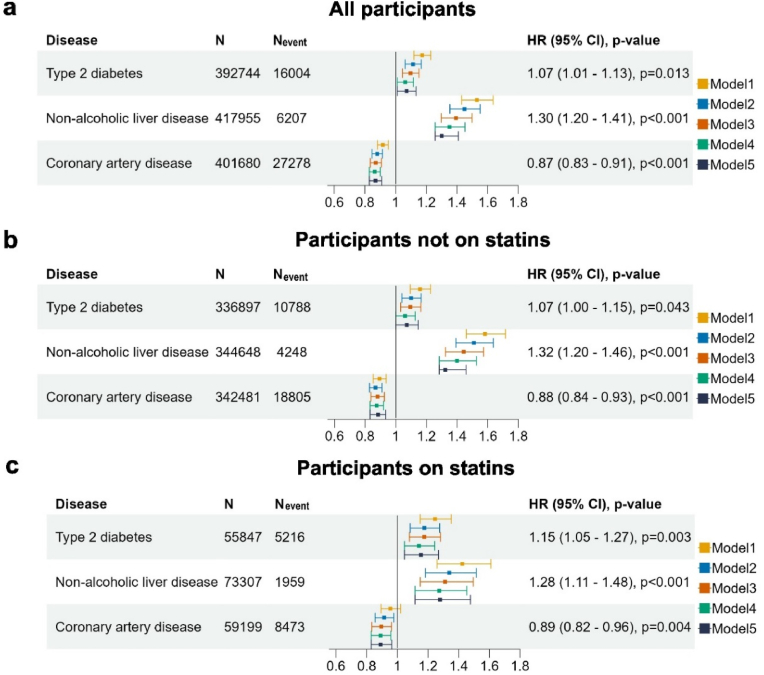
Forest plot illustrating the unadjusted and adjusted hazard ratios of very low Lp(a) concentrations at baseline with the development of T2D, NAFLD and CAD. Hazard ratios for diseases in **(a)** all participants **(b)** participants not using statin and **(c)** participants using statin in model 5. Model 1: adjusted for age and sex; Model 2: Model 1 + body mass index and hypertension; Model 3: Model 2 + low-density lipoprotein cholesterol and high-density lipoprotein cholesterol; Model 4: Model 3 + triglycerides; Model 5: Model 4 + alcohol consumption in UK Units (8g pure alcohol) per week at baseline visit. Hazard ratios (HR) with 95 % confidence intervals (CI) and p-values of Model 5 are shown for new-onset T2D, NAFLD and CAD. N: Number of participants, N_event:_ number of events.

In all participants combined, Cox regression analyses showed that very low Lp(a) concentrations at baseline showed trend of increased risk of T2D but the association was not statistically significant after considering multiple testing corrections (fully adjusted HR:1.07; 95 % CI 1.01–1.13; *p* = 0.013). We observed increased risk of NAFLD (fully adjusted HR:1.30; 95 % CI 1.20–1.41; *p* < 0.001), in contrast to the decreased risk of CAD (fully adjusted HR 0.87; 95 % CI 0.83–0.91; *p* < 0.001) (Fig. 2). This inverse association with new-onset T2D was more prominent in participants using statin at baseline (age- and sex-adjusted interaction *p* = 0.04). Additional adjustments attenuated the association and it lost significance after further adjustment for triglycerides, both among participants on statin (HR 1.15; 95 % CI 1.05–1.27; *p* = 0.003) and in those not on statin (HR 1.07; 95 % CI 1.00–1.15; *p* = 0.043) respectively (Table S4). The association with NAFLD remained statistically significant in the fully adjusted model, which adjusted for age, sex, BMI, hypertension, LDL-C, HDL-C, triglycerides and alcohol consumption, in the full cohort (Fig. 2). Subgroup analysis by statin use revealed comparable point estimates of the HR (Table S5). Reverse trends were observed in participants with very high Lp(a) concentrations (Fig. S2).

We continued by modeling inverse rank normalized Lp(a) concentrations among participants whose Lp(a) concentrations were within the reportable range (N = 371,430). We observed smaller point estimates with neither association with diabetes nor NAFLD being significant in the fully adjusted Model 5 (Fig. S3). Given the ethnic difference in Lp(a), we assessed the associations with new-onset T2D (Table S6) and NAFLD (Table S7) by ethnic groups in participants not on statin. We found no evidence of interaction effects between either disease and White ethnicity (age and sex adjusted interaction *p* = 0.149 for T2D and *p* = 0.456 for NAFLD, respectively).

**Fig. 3:**
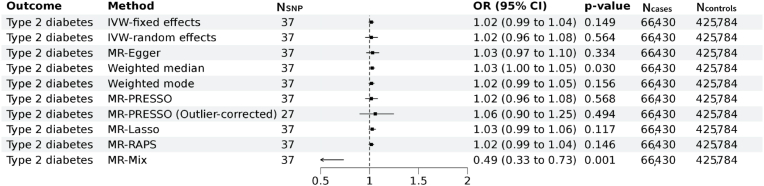
**Mendelian randomization assessing the genetic association of Lp(a) with Type 2 diabetes.** Forest plot of the Mendelian randomization results of Lp(a) with type 2 diabetes. Results of the MR IVW-fixed effects, IVW-random effects, MR Egger, weighted median, weighted mode, MR-PRESSO, MR Lasso, MR-Raps and MR-mix are provided. Odds ratios and 95 % confidence intervals are shown. We found evidence for vertical pleiotropy in the current analyses (I^2^ index 84.1 % (CI = 78.9–88.0); Cochran's Q = 230, degrees of freedom (df) = 36, *p* = 2.4 × 10^−29^; Q-Q’ = 16, df = 1, *p* = 8 × 10^−5^; MR-Egger intercept −0.0049 ± 0.0030, *p* = 0.116). There was no evidence for weak instrument bias in the MR-Egger regression (I^2^_GX_ = 0.99).MR: Mendelian randomization, IVW = inverse variance weighted, MR-PRESSO: Mendelian Randomization Pleiotropy RESidual Sum and Outlier, MR-RAPS: Mendelian randomization using the robust adjusted profile score, OR = odds ratio, CI = Confidence interval.

**Fig. 4:**
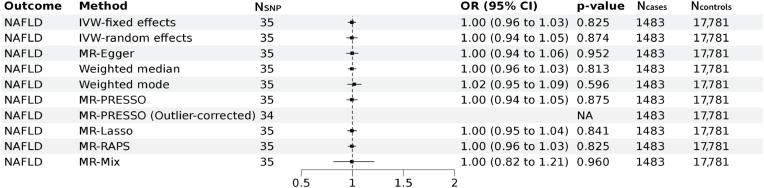
**Mendelian randomization assessing the genetic association of Lp(a) with NAFLD.** Forest plot of the Mendelian randomization results of Lp(a) with non-alcoholic fatty liver disease (NAFLD). Results of the MR IVW-fixed effects, IVW-random effects, MR Egger, weighted median, weighted mode, MR-PRESSO, MR Lasso, MR-Raps and MR-mix are provided. Odds ratios and 95 % confidence intervals are shown. We found evidence for horizontal pleiotropy in the current analyses (I^2^ index 48.6 % (CI = 23.8–65.3); Cochran's Q = 66, degrees of freedom (df) = 34, *p* = 0.0008; Q-Q’ = 0.19, df = 1, *p* = 0.661; MR-Egger intercept −0.0021 ± 0.0069, *p* = 0.759). There was no evidence for weak instrument bias in the MR-Egger regression (I^2^_GX_ = 0.99).MR: Mendelian randomization, IVW = inverse variance weighted, MR-PRESSO: Mendelian Randomization Pleiotropy Residual Sum and Outlier, MR-RAPS: Mendelian randomization using the robust adjusted profile score, OR = odds ratio, CI = Confidence interval.

We next performed two-sample MR analyses to test whether genetically predicted Lp(a) is associated with T2D and NAFLD with GWAS summary statistics from external cohorts namely DIAMANTE for T2D and EPos for NAFLD respectively. A total of 37 and 35 genetic instruments for Lp(a) were available for the MR analyses in the DIAMANTE and EPoS GWAS repositories. We did not find evidence for an association of genetically predicted Lp(a) with T2D (IVW-fixed effects OR:1.02; 95 % CI: 0.99–1.04, *p* = 0.149) (Fig. 3). Sensitivity analyses indicated presence of balanced vertical pleiotropy with non-significant intercept (−0.0049 ± 0.0030, *p* = 0.116) in the MR-Egger model. Estimates by IVW-random effects (OR:1.02; 95 % CI: 0.96–1.08, *p* = 0.564) were consistent with most methods except for the MR-Mix model. We furthermore found no evidence for an association of genetically predicted Lp(a) with NAFLD (IVW-fixed effects OR:1.00; 95 % CI: 0.96–1.03, *p* = 0.825) with evidence for horizontal pleiotropy in the sensitivity analyses (Fig. 4). Estimates in the sensitivity analyses were consistent with IVW-fixed effects models.

## 4 Discussion

Previous work equivocally suggested that higher Lp(a) concentration may be associated with a lower risk of diabetes [1,28]. Accordingly, the EAS consensus statement published in 2022 has suggested that there is an unmet need to dissipate whether a potential association of very low levels of Lp(a) with higher risk of diabetes development is causal [1]. By using an extended biomarker dataset in over 450,000 UK Biobank participants, we confirmed epidemiological observation of the higher risk of T2D in individuals with very low Lp(a) concentrations (<3.8 nmol/L) contrasting its protective effect on CVDs. Additional analyses on participants with very high Lp(a) concentrations (>189 nmol/L) as well as on participants with Lp(a) measurements within the reportable range supported the robust link between Lp(a) and CAD. In comparison, the reverse associations for T2D and NAFLD showed consistent trends but were less prominent. The association for T2D was no longer significant after adjusting for other cardiometabolic risk factors. In contrast, association between low Lp(a) and increased NAFLD risk remained in the fully adjusted model.

Given previous report of increased risk of new-onset T2D with statin use [14], we further assessed the association in subgroups stratified by statin. Notably we observed the similar protective effect for CAD in both statins group and non-statins group in participant with very low Lp(a); we found that the risk of diabetes was enhanced in individuals on statins while the risk of NAFLD was attenuated in the context of statin use. We noted that only the associations for NAFLD remained statistically significant in both groups in the fully adjusted model. The statistical interactions were not significant as assessed by a product term in the Cox model.

Our results from the two sample MR analyses using 37 lead SNPs, which included rs10455872 (a common intronic SNP strongly associated with the Lp(a) concentration in White individuals), as genetic instruments, did not support a causal relationship between Lp(a) and T2D nor NAFLD. Our results extend findings of previous work which solely used rs10455872 [29]. Sensitivity analyses suggested presence of pleiotropy in the association between Lp(a) with both traits though we found the estimates remained consistent in the sensitivity analyses. Association between Lp(a) isoform size and risk of T2D were also reported [30] though we were unable to investigate the role of Lp(a) isoform size as these data are not available in the UK Biobank, which is among the limitations of the current study.

Several studies have implicated a role of triglyceride-rich lipoproteins in the metabolism of Lp(a) [8,31,32], whereas conversely genetically predicted Lp(a) may influence very low density lipoprotein (VLDL) metabolism [33]. These findings corroborate to our study on the association of Lp(a) with new-onset T2D and NAFLD in the context of statin use, and to adjust for triglycerides when examining the association of Lp(a) with T2D and NAFLD. The inverse associations of NAFLD in Cox regression and absence of causal link in MR analyses found in our study of the UK Biobank also aligned with a recent study which proposed Lp(a) as a biomarker for liver damage [34]. Experimental studies are needed to study the intricate interactions between Lp(a) and VLDL as well as its involvement in the associations with diabetes and non-alcoholic liver disease reported in observational studies, and to validate the biological mechanisms behind.

Strengths of the current study include the large dataset from the UK Biobank obviating the need to harmonize plasma Lp(a) measurements. However, most UK Biobank participants are White, precluding to test robust interactions of Lp(a) with ethnicity on diabetes development. This is relevant because the distribution of plasma Lp(a) varies considerably among ethnicities with a median concentration being much higher in Black vs, White individuals [1].

## 5 Conclusions

Using prospective data of the UK Biobank repository, very low association for Lp(a) concentrations with T2D was modified by statin usage. We found no evidence for an association between genetically predicted Lp(a) and T2D or NAFLD. Our current findings in both longitudinal analysis and MR analysis do not substantiate any concerns in exacerbated T2D risk by aggressive Lp(a) lowering therapy.

## 6 Ethics approval and consent to participate

The UK Biobank has ethical approval from North West - Haydock Research Ethics Committee (REC reference: 16/NW/0274). All participants have given informed consent.

## 7 Availability of data and materials

The data that support the findings of this study are available from the corresponding authors upon reasonable request. Source data may be requested from the UK biobank.

## Competing interests

M.W.Y. is currently employed by and holds stock in GSK plc; this work was conducted before employment by GSK. N.V. is a full-time employee of Regeneron Pharmaceutical Inc. and receives stock options and restricted stock units as compensation. Other authors declare no competing interests.

## Funding

None.

## 8 CRediT authorship contribution statement

M.W.Y, M.A.S., R.P.F.D., P.v.d.H: Conceptualization; M.W.Y, M.A.S., Y.J.v.d.V, N.V.: Methodology, Software; M.W.Y, M.A.S.: Investigation; R.P.F.D, P.v.d.H: Supervision; M.W.Y:Writing - Original Draft, review & editing; M.A.S., Y.J.v.d.V, N.V., R.P.F.D., P.v.d.H: Writing – Critical review & Editing.

## Declaration of competing interest

The authors declare the following financial interests/personal relationships which may be considered as potential competing interests:

M.W.Y. is currently employed by and holds stock in GSK; this work was conducted before employment by GSK. N.V. is a full-time employee of Regeneron Pharmaceutical Inc. and receives stock options and restricted stock units as compensation. Other authors declare no competing interests.

## References

[1] Kronenberg F., Mora S., Stroes E.S.G. (2022). Lipoprotein(a) in atherosclerotic cardiovascular disease and aortic stenosis: a European Atherosclerosis Society consensus statement. Eur Heart J.

[2] Dullaart R.P.F. (2020). Lipoprotein(a): the renaissance of an enigmatic lipoprotein. J Clin Endocrinol Metab.

[3] Said M.A., Yeung M.W., Van De Vegte Y.J. (2021). Genome-Wide association study and identification of a protective missense variant on lipoprotein(a) concentration: protective missense variant on lipoprotein(a) concentration - brief report. Arterioscler Thromb Vasc Biol.

[4] Jung I., Kwon H., Park S.E. (2020). Serum lipoprotein(a) levels and insulin resistance have opposite effects on fatty liver disease. Atherosclerosis.

[5] Kwok R., Choi K.C., Wong G.L.-H. (2016). Screening diabetic patients for non-alcoholic fatty liver disease with controlled attenuation parameter and liver stiffness measurements: a prospective cohort study. Gut.

[6] De Silva N.M.G., Borges M.C., Hingorani A.D. (2019). Liver function and risk of type 2 diabetes: bidirectional mendelian randomization study. Diabetes.

[7] Liu Z., Zhang Y., Graham S. (2020). Causal relationships between NAFLD, T2D and obesity have implications for disease subphenotyping. J Hepatol.

[8] Nestel P., Loh W.J., Ward N.C., Watts G.F. (2022). New horizons: revival of lipoprotein (a) as a risk factor for cardiovascular disease. J Clin Endocrinol Metab.

[9] Sudlow C., Gallacher J., Allen N. (2015). UK biobank: an open access resource for identifying the causes of a wide range of complex diseases of middle and old age. PLoS Med.

[10] Verweij N., Haas M.E., Nielsen J.B. (2022). Germline mutations in CIDEB and protection against liver disease. N Engl J Med.

[11] Yeung M.W., van der Harst P., Verweij N. (2022). Ukbpheno v1.0: an R package for phenotyping health-related outcomes in the UK Biobank. STAR Protoc.

[12] UK Biobank (2011). Blood sample collection, processing and transport. https://biobank.ndph.ox.ac.uk/ukb/ukb/docs/Bloodsample.pdf.

[13] Fry D., Almond R., Moffat S., Gordon M., Singh P. (2019).

[14] Sattar N., Preiss D., Murray H.M. (2010). Statins and risk of incident diabetes: a collaborative meta-analysis of randomised statin trials. Lancet.

[15] Mahajan A., Spracklen C.N., Zhang W. (2022). Multi-ancestry genetic study of type 2 diabetes highlights the power of diverse populations for discovery and translation. Nat Genet.

[16] Anstee Q.M., Darlay R., Cockell S. (2020). Genome-wide association study of non-alcoholic fatty liver and steatohepatitis in a histologically characterised cohort. J Hepatol.

[17] Hemani G., Zheng J., Elsworth B. (2018). The MR-Base platform supports systematic causal inference across the human phenome. Elife.

[18] Burgess S., Foley C.N., Allara E., Staley J.R., Howson J.M.M. (2020). A robust and efficient method for Mendelian randomization with hundreds of genetic variants. Nat Commun.

[19] Qi G., Chatterjee N. (2019). Mendelian randomization analysis using mixture models for robust and efficient estimation of causal effects. Nat Commun.

[20] Verbanck M., Chen C.-Y., Neale B., Do R. (2018). Detection of widespread horizontal pleiotropy in causal relationships inferred from Mendelian randomization between complex traits and diseases. Nat Genet.

[21] Zhao Q., Wang J., Hemani G., Bowden J., Small D.S. (2019).

[22] Bowden J., Davey Smith G., Burgess S. (2015). Mendelian randomization with invalid instruments: effect estimation and bias detection through Egger regression. Int J Epidemiol.

[23] Rees J.M.B., Wood A.M., Dudbridge F., Burgess S. (2019). Robust methods in Mendelian randomization via penalization of heterogeneous causal estimates. PLoS One.

[24] Palmer T.M., Lawlor D.A., Harbord R.M. (2012). Statistical methods in medical research.

[25] Greco M.F.D., Minelli C., Sheehan N.A., Thompson J.R. (2015). Detecting pleiotropy in Mendelian randomisation studies with summary data and a continuous outcome. Stat Med.

[26] Bowden J., Del Greco M.F., Minelli C., Davey Smith G., Sheehan N., Thompson J. (2017). A framework for the investigation of pleiotropy in two-sample summary data Mendelian randomization. Stat Med.

[27] Bowden J., Del Greco M.F., Minelli C., Davey Smith G., Sheehan N.A., Thompson J.R. (2016). Assessing the suitability of summary data for two-sample Mendelian randomization analyses using MR-Egger regression: the role of the I2 statistic. Int J Epidemiol.

[28] Månsson M., Kalies I., Bergström G. (2014). Lp(a) is not associated with diabetes but affects fibrinolysis and clot structure ex vivo. Sci Rep.

[29] Ye Z., Haycock P.C., Gurdasani D. (2013). The association between circulating lipoprotein(a) and type 2 diabetes: is it causal?. Diabetes.

[30] Mu-Han-Ha-Li D.-L.-D.-E., Zhai T.-Y., Ling Y., Gao X. (2018). LPA kringle IV type 2 is associated with type 2 diabetes in a Chinese population with very high cardiovascular risk. J Lipid Res.

[31] Moriarty P.M., Varvel S.A., Gordts P.L.S.M., McConnell J.P., Tsimikas S. (2017). Lp(a) mass levels increase significantly according to APOE genotype: an analysis of 431,239 patients. Arterioscler Thromb Vasc Biol.

[32] Croyal M., Blanchard V., Ouguerram K. (2020). VLDL (very-low-density lipoprotein)-Apo e (Apolipoprotein E) may influence Lp(a) (Lipoprotein [a]) synthesis or assembly. Arterioscler Thromb Vasc Biol.

[33] Kettunen J., Demirkan A., Würtz P. (2016). Genome-wide study for circulating metabolites identifies 62 loci and reveals novel systemic effects of LPA. Nat Commun.

[34] Meroni M., Longo M., Lombardi R. (2021). Low lipoprotein(a) levels predict hepatic fibrosis in patients with nonalcoholic fatty liver disease. Hepatol Commun.

