# Supplementary material for: Associations of very low Lipoprotein(a) levels with risks of new-onset diabetes and non-alcoholic liver disease
Source: Atheroscler Plus. 2024 Jul 11;57:19–25. doi: 10.1016/j.athplu.2024.07.001 (PMC11300918; doi:10.1016/j.athplu.2024.07.001)
Supplement: Multimedia component 1 [file mmc1.pdf]

Supplementary Material

Very Low Lipoprotein(a) Levels Are Associated With Higher Risks Of New-Onset Diabetes And Non-Alcoholic Liver Disease

Ming Wai Yeung[1], M. Abdullah Said[1], Yordi J. van de Vegte[1],Niek Verweij[1], Robin P.F. Dullaart[3*], Pim van der Harst[1,2*]

Author affiliations:
[1] Department of Cardiology University of Groningen, University Medical Center Groningen, , 9700 RB Groningen, the Netherlands
[2]Department of Cardiology, Division of Heart & Lungs, University Medical Center Utrecht, University of Utrecht, Utrecht, the Netherlands
[3]Department of Endocrinology, University of Groningen, University Medical Center Groningen, Groningen, The Netherlands.

*Contributed equally

Contents:

Tables S1-S7

Figures S1-S3 including figure legends

Table S1. Data fields used to identify lifestyle information in the UK Biobank

Lifestyle factor	Data field (value)
Smoking status (active smoker)	22506 (111)<br>1239(1)<br>20116(2)
Weekly alcohol consumption*	1568,4407<br>1578,4418<br>5364,4462<br>1588,4429<br>1598,4440<br>1608,4451

* Average weekly alcohol consumption data were collected from both monthly-average and weekly-average questions during the touchscreen interview. Alcohol consumptions were computed as the sum of all alcohol types converted to UK units and the maximum weekly estimate between the two answers were taken forward to represent the weekly alcohol consumption of the participant.

Table S2. Clinical codes used to define disease and medication in the UK Biobank

Outcome	ICD-9 / ICD-10	OPCS-4	Self-reported fields	READ2	CTV3	BNF	DMD
Aortic stenosis	I350, I352	K621, K622, K623	20002(1490)	G5411, G5413, G5414, G5415	G5411, G5413, G5414, X2011, Xa0Ct, X2015		
Coronary artery disease	414, 410, 412; I24, I25, Z955, I21, I22, I23, I252, Z951, Z955	K40, K41, K42, K43, K44, K45, K46, K49, K50, K75	20002(1075), 20004(1070, 1095, 1523),6150(1)	G34y1, G34.., G3..., ZV45L, G34z0, ZV458, 793G., 79280, 79281, 79282, 7928y, 7928z, 79292, 7929y, 7929z, 792.., 7A547, 793Gy, 793Gz, 79283	G34y1, XE0WG, XE2uV, XaC1g, XaG1Q, XaQiY, ZV458, G34.., X200b, Xa1dP, XaLgU, 79280, 79281, 79282, 7928y, 7928z, 79292, 7929y, 7929z, X00tT, X013N, XE0Em, XaLgZ, XaLga, XaMKE		
Diabetes*	250; E10-E14		20002(1220,1222 ,1223), 2443(1),6153(3),6 177(3)	C10..	C10.., X40J4, X40J5, X40J6, X40JJ, X40Jj, X40Jl		
Heart failure	428; I50, I110, I130, I132, Z941, T862	K02	20002(1076), 20004(1098)	14S3., G2101, G2111, G21z1, G232., G234., G58.., G5800, G5802, G5803, G5810, G582., SP084, SP111, G581., 1O1.., G583., ZV421	14S3., G2101, G2111, G21z1, G232., G234., G58.., G5800, G5802, G5803, G5810, G582., SP084, X202k, X202v, X202w, XE2QG, XaIpn, XaWyi, ZV421, X00y3		
Hypertension	402, 403, 404, 405, 401; I11, I12, I13, I15, O10, I10		20002(1065, 1072),6150(4),61 77(2),6153(2),200 03	G21.., G220., G221., G23.., G24.., G240., G240z, G241., G241z, G24z., L12.., G22.., G22z., G2z.., G2y.., G21z0, G20.., G20z., x01QX	G21.., G220., G221., G23.., G24.., G240., G240z, G241., G241z, G24z., L12.., XE0Uf, XE0Ug, G2z.., G2y.., Xa0lt, Xa3fQ, Xa0kX, XE0Uc, x01QX		
Non-alcoholic liver disease	K721, K740, K741, K742, K746, K758, K760			J61y4, J61y1, J61y3, J61y7, J62y., J61y6, J61y9, J61y8	J61y1, J61y7, X307C, X307W, X307b, X307v, XE0b5, XaQIT, J61y8, J61y9, J625.		
Peripheral artery disease	4439; I739	L37, L381, L383, L384, L391, L392, L395, L48, L49, L50, L51, L52, L53, L541, L542, L544, L56, L57, L58, L59, L60, L62, L631, L632, L635, L638, L639, L653	20002(1087, 1067), 20004(1102, 1103, 1108)	G73., 7A26., 7A270, 7A271, 7A276, 7A277, 7A27C, 7A27E, 7A279, 7A27B, 7A27D, 7A280, 7A281, 7A283, 7A28D, 7A28C, 7A28G, 7A40., 7A41., 7A42., 7A43., 7A440, 7A441, 7A45., 7A46., 7A47., 7A48., 7A4A., 7A4B0, 7A4B1, 7A4B2, 7A4B3, 7A4B9, 7A4By, 7A4Bz, 7A502, 7A2., 7A443, 7A4B., 7A433	Xa0lV, G73z., XE0VP, XE0F9, 7A270, 7A276, 7A27C, 7A280, 7A283, XaMMk, 7A26., X013S, Xa7pt, XaCLU, X015N, 7A277, 7A281, 7A284, Xa9Iv, X013T, XE0FJ, X016D, X015h, XE0FK, X015H, XE0FN, XM1Ih, XE0FQ, 7A440, 7A441, XaDmi, 7A40., 7A41., 7A42., 7A43., 7A443, X013i, X013k, X013m, X016n, X016o, X70XH, XE0FI, XaDmh, XaG1G, X015g, X015f, XM1M6, XE0FV, X015d, XM1Ik, XM1Im, XE0FW, XM1In, XM1Il, X015X, X015b, X015c, XE0FX, X015Y, X015R, X015a, XE0FY, X015V, X015U, X015T, X015W, XM1Ir, XM1Is, XE0FZ, 7A45., 7A46., 7A47., 7A48., 7A49., 7A4A., X013o, X013p, X013q, X013r, X014V, X014W, 7A502, 7A4Bz, 7A4By, XaMNe, 7A4B2, 7A4B0, XM1Iv, XM1It, XM1Iw, XM1Iq, XM1KK, XaIzW, XaE0L, Xa0Fu, Xa0Fr, XM1J1, XM1Iz, XM1Iy, XM1Ix, XE0Fb, X016p, X0159, X015O, 7A279, 7A27B, 7A27D, 7A28C, 7A28D, Xa7pv, Xa7y1, XaCLY, X015e, XM1Io, X015Z, X015S, XM1Ip, XM1Iu, 7A4B1, 7A4B3, X013u, X014h, X015C, XM1J0, Xa0Fz		

Outcome	ICD-9 / ICD-10	OPCS-4	Self-reported fields	READ2	CTV3		
Stroke	430,431,434,435,436,4371, 3361, 36231, 36232, 4329, 43301, 43311, 43321, 43331, 43381, 43391; I60, I61, I63, I64, I65, I629, I678, I690, I693, G45, G951, H341, H342, S066	A052, A053, A054, L343, L351, L353,	20002(1081,1082,1086,1491,1583),6150(3)	F4236,G65zz,G64z4,G66..,G64..,F4232,G65..,F4238,G64z.,F4239,G668.,G64z2,G60..,F423.,G679.,G61..,G62..,G655.,G673.,G678.,G667.,G623.,G665.,G61X.,G64z1,G622.,G6400,G650.,G64z3,G613.,G605.,G652.,G662.,G621.,G61z.,G664.,G600.,G6740,G60z.,G640.,G663.,G61X0,G6760,G6732,G6733,G6772,F4231,G641.,G671.,G602.,G67z.,70043,G614.,G604.,F423z,G610.,Gyu6F,G64z0,G61X1,G656.,G660.,F4230,G62z.,G65z.,70041,G6510,G676.,G611.,G6731,G617.,G666.,G612.,G6410,G619.,G6711,G657.	X00D1,XE0VK,XaBEC,XE2aB,G65z.,XE0Wy,F4231,Xa1uW,X00D7,Xa0kZ,Xa00J,G664.,XE0VF,G62..,G65y.,G677.,Xa00I,XaBED,G676.,X00D5,XM0rV,G62z.,G61z.,X00D6,G65z0,G673.,Xa01h,X00DV,Xa00K,X00db,Xa01l,Xa3fV,X00DU,XaEIh,Xa01k,G611.,G663.,X00DR,G621.,G6711,70043,X00Dg,G60z.,G6y..,G675.,G612.,Xa84g,Xa01i,G6760,Xa1hE,G671.,G67..,G614.,Xa01j,XaBM4,70041,X00DN,G613.,X00DO,XaBM5,Gyu60,G610.,G601.,Gyu6F,G683.,G6772,Xa0N7,F1611,G6771,Gyu6E		
Use of statin			20003(1140861958,1140881748,1140910652,1140910654,1141188146,1141195196,1141200040,1140861970,1140864592,1141146138,1141146234,1141192410,1141192414,1140888594,1140888648,1140910632)	bxd,bxe,bxg,bxi,bxj,bxk,x01R2,x01R3	bxd,bxe,bxg,bxi,bxj,bxk,x01R2,x01R3	02.12.04.00,02.12.02.00, 0212000B0AAAAAA, 0212000B0AAABAB, 0212000B0AAACAC, 0212000C0AAAAAA, 0212000C0AAABAB, 0212000C0AAACAC, 0212000M0AAAAAA, 0212000M0AAABAB, 0212000X0AAAAAA, 0212000X0AAABAB, 0212000X0AAADAD, 0212000Y0AAAAAA, 0212000Y0AAABAB, 0212000Y0AAADAD, 02120200, 02.12.02.00.00, 02120400, 02.12.04.00.00	134489001, 319996000, 319997009, 320000009, 320006003, 320012008, 320013003, 320014009, 320022002, 320023007, 320025000, 320029006, 320030001, 320031002, 320035006, 320036007, 320037003, 320041004, 408024009, 408036003, 408037007, 409108001, 414177002, 414178007, 414179004, 4580311000001109, 19722411000001106, 19722511000001105, 20528511000001106, 20528611000001105, 240705001000027108, 240715001000027105, 299275001000027104

Variable definitions constructed using ICD-9, ICD-10, OPCS-4, READ2 and CTV3 codes as well as self-report data fields with disease- or procedure-specific codes between brackets are shown. *: We additionally removed participants with glycated haemoglobin (HbA1c) ≥ 48 mmol/mol at baseline were removed from the Cox regression analysis. Abbreviations: CTV3, Clinical Terms Version 3; ICD, International Classification of Diseases; OPCS, Office of Population, Censuses and Surveys: Classification of interventions and Procedure.

Table S3. Censoring dates for participants by region

Region	Censoring date
England	30th September 2021
Scotland	31st July 2021
Wales	28th February 2018

Table S4. Multivariable Cox proportional hazard analysis of Lp(a) concentration on type 2 diabetes

Model	Covariates	HR	95% CI	*p*	Model	HR	95% CI	*p*
all participants								
		very low Lp(a)				very high Lp(a)		
Model 1	Age, Sex	1.17	1.12 - 1.23	<0.001	Model 1	1.07	1.00 - 1.13	0.028
Model 2	Model 1 + BMI and hypertension	1.11	1.06 - 1.17	<0.001	Model 2	1.00	0.95 - 1.06	0.867
Model 3	Model 2 + LDL-C + HDL-C	1.10	1.04 - 1.15	<0.001	Model 3	1.06	1.00 - 1.12	0.072
Model 4	Model 3 + triglycerides	1.06	1.01 - 1.12	0.016	Model 4	1.08	1.02 - 1.15	0.009
Model 5	Model 4 + alcohol consumption	1.07	1.01 - 1.13	0.013	Model 5	1.10	1.03 - 1.18	0.004
participants not on statin								
		very low Lp(a)				very high Lp(a)		
Model 1	Age, Sex	1.16	1.10 - 1.23	<0.001	Model 1	0.91	0.84 - 0.98	0.019
Model 2	Model 1 + BMI and hypertension	1.10	1.04 - 1.16	<0.001	Model 2	0.90	0.83 - 0.98	0.015
Model 3	Model 2 + LDL-C + HDL-C	1.10	1.03 - 1.16	0.003	Model 3	0.94	0.86 - 1.02	0.125
Model 4	Model 3 + triglycerides	1.06	1.00 - 1.13	0.055	Model 4	0.97	0.89 - 1.05	0.442
Model 5	Model 4 + alcohol consumption	1.07	1.00 - 1.15	0.043	Model 5	0.99	0.90 – 1.09	0.809
participants on statin								
		very low Lp(a)				very high Lp(a)		
Model 1	Age, Sex	1.25	1.15 - 1.35	<0.001	Model 1	0.94	0.86 - 1.02	0.112
Model 2	Model 1 + BMI and hypertension	1.18	1.08 - 1.28	<0.001	Model 2	0.95	0.88 - 1.03	0.253
Model 3	Model 2 + LDL-C + HDL-C	1.18	1.08 - 1.28	<0.001	Model 3	0.99	0.91 - 1.08	0.771
Model 4	Model 3 + triglycerides	1.14	1.05 - 1.24	0.003	Model 4	1.02	0.93 - 1.11	0.711
Model 5	Model 4 + alcohol consumption	1.15	1.05 - 1.27	0.003	Model 5	1.02	0.93 – 1.12	0.745

Very low Lp(a): Lp(a) <3.8 nmol/L; very high Lp(a): Lp(a) >189 nmol/L. BMI: body mass index, CI: confidence interval, HDL-C: high density lipoprotein cholesterol, HR: hazard ratio, LDL-C: low-density lipoprotein cholesterol.

Table S5. Multivariate logistic regression of Lp(a) concentration on non-alcoholic liver disease

Model	Covariates	HR	95% CI	*p*	Model	HR	95% CI	*p*
all participants								
		very low Lp(a)				very high Lp(a)		
Model 1	Age, Sex	1.53	1.43 - 1.64	<0.001	Model 1	0.89	0.80 - 0.98	0.023
Model 2	Model 1 + BMI and hypertension	1.45	1.35 - 1.55	<0.001	Model 2	0.84	0.76 - 0.93	0.001
Model 3	Model 2 + LDL-C + HDL-C	1.39	1.30 - 1.50	<0.001	Model 3	0.89	0.80 - 0.99	0.031
Model 4	Model 3 + triglycerides	1.35	1.26 - 1.45	<0.001	Model 4	0.91	0.82 - 1.01	0.084
Model 5	Model 4 + alcohol consumption	1.30	1.20 - 1.41	<0.001	Model 5	0.88	0.79 – 0.99	0.038
participants not on statin								
		very low Lp(a)				very high Lp(a)		
Model 1	Age, Sex	1.58	1.46 - 1.72	<0.001	Model 1	0.78	0.68 - 0.90	<0.001
Model 2	Model 1 + BMI and hypertension	1.51	1.39 - 1.64	<0.001	Model 2	0.78	0.68 - 0.90	<0.001
Model 3	Model 2 + LDL-C + HDL-C	1.44	1.32 - 1.57	<0.001	Model 3	0.85	0.73 - 0.98	0.029
Model 4	Model 3 + triglycerides	1.40	1.28 - 1.53	<0.001	Model 4	0.88	0.76 - 1.02	0.083
Model 5	Model 4 + alcohol consumption	1.32	1.20 - 1.46	<0.001	Model 5	0.83	0.71 – 0.98	0.029
participants on statin								
		very low Lp(a)				very high Lp(a)		
Model 1	Age, Sex	1.42	1.26 - 1.61	<0.001	Model 1	0.80	0.69 - 0.92	0.002
Model 2	Model 1 + BMI and hypertension	1.34	1.18 - 1.51	<0.001	Model 2	0.83	0.71 - 0.96	0.012
Model 3	Model 2 + LDL-C + HDL-C	1.31	1.15 - 1.50	<0.001	Model 3	0.85	0.73 - 1.00	0.047
Model 4	Model 3 + triglycerides	1.27	1.12 - 1.45	<0.001	Model 4	0.87	0.75 - 1.02	0.084
Model 5	Model 4 + alcohol consumption	1.28	1.11 - 1.48	<0.001	Model 5	0.87	0.73 – 1.03	0.105

Very low Lp(a): Lp(a) <3.8 nmol/L; very high Lp(a): Lp(a) >189 nmol/L. BMI: body mass index, CI: confidence interval, HDL-C: high density lipoprotein cholesterol, HR: hazard ratio, LDL-C: low-density lipoprotein cholesterol.

Table S6. Association of very low Lp(a) concentration with diabetes stratified by self-reported ethnic groups

Ethnic group	Model	Covariates	HR	95% CI	*p*	N	N_{event}
White							
	Model 1	Age, Sex	1.24	1.17-1.31	<0.001	318,960	9,493
	Model 2	Model 1 + BMI and hypertension	1.17	1.10-1.24	<0.001	317,477	9,420
	Model 3	Model 2 + LDL-C + HDL-C	1.17	1.10-1.24	<0.001	289,203	8,591
	Model 4	Model 3 + triglycerides	1.13	1.07-1.21	<0.001	289,169	8,589
	Model 5	Model 4 + alcohol consumption	1.14	1.07-1.23	<0.001	247,084	6,790
Asian							
	Model 1	Age, Sex	1.08	0.75-1.56	0.671	6,634	554
	Model 2	Model 1 + BMI and hypertension	0.97	0.67-1.41	0.882	6,579	542
	Model 3	Model 2 + LDL-C + HDL-C	1.06	0.71-1.57	0.791	5,982	489
	Model 4	Model 3 + triglycerides	1.04	0.70-1.55	0.828	5,982	489
	Model 5	Model 4 + alcohol consumption	1.06	0.69-1.61	0.795	5,150	426
Black							
	Model 1	Age, Sex	1.14	0.51-2.55	0.756	4,844	402
	Model 2	Model 1 + BMI and hypertension	1.26	0.56-2.83	0.573	4,775	395
	Model 3	Model 2 + LDL-C + HDL-C	0.68	0.22-2.12	0.507	4,388	364
	Model 4	Model 3 + triglycerides	0.51	0.16-1.62	0.254	4,387	364
	Model 5	Model 4 + alcohol consumption	0.40	0.10-1.66	0.207	3,622	279
Mixed							
	Model 1	Age, Sex	1.49	0.72-3.09	0.283	2,097	85
	Model 2	Model 1 + BMI and hypertension	1.59	0.77-3.31	0.211	2,084	85
	Model 3	Model 2 + LDL-C + HDL-C	1.31	0.57-3.03	0.524	1,893	76
	Model 4	Model 3 + triglycerides	N/A				
	Model 5	Model 4 + alcohol consumption	N/A				
Other							
	Model 1	Age, Sex	0.98	0.62-1.55	0.930	4,362	254
	Model 2	Model 1 + BMI and hypertension	1.01	0.64-1.60	0.967	4,288	250
	Model 3	Model 2 + LDL-C + HDL-C	0.93	0.56-1.52	0.760	3,913	231
	Model 4	Model 3 + triglycerides	0.85	0.52-1.41	0.538	3,913	231
	Model 5	Model 4 + alcohol consumption	1.05	0.58 -1.91	0.875	2,933	149

Very low Lp(a): Lp(a) <3.8 nmol/L. Models with <10 event-per-variable were not reported. BMI: body mass index, CI: confidence interval, HDL-C: high density lipoprotein cholesterol, HR: hazard ratio, LDL-C: low-density lipoprotein cholesterol, N: Number of participants in group, N_{event}: number of events for the group.

Table S7. Association of very low Lp(a) concentration with non-alcoholic liver disease stratified by self-reported ethnic groups

Ethnic group	Model	Covariates	HR	95% CI	*p*	N	N_{event}
White							
	Model 1	Age,Sex	1.59	1.46-1.73	<0.001	325,393	3,963
	Model 2	Model 1 + BMI and hypertension	1.50	1.38-1.63	<0.001	323,847	3,936
	Model 3	Model 2 + LDL-C + HDL-C	1.45	1.33-1.58	<0.001	295,025	3,595
	Model 4	Model 3 + triglycerides	1.41	1.29-1.54	<0.001	294,988	3,594
	Model 5	Model 4 + alcohol consumption	1.34	1.21-1.47	<0.001	251,758	2,928
Asian							
	Model 1	Age,Sex	1.56	0.79-3.08	0.204	7,239	110
	Model 2	Model 1 + BMI and hypertension	1.47	0.74-2.91	0.273	7,168	107
	Model 3	Model 2 + LDL-C + HDL-C	1.07	0.47 - 2.46	0.870	6,524	98
	Model 4	Model 3 + triglycerides	1.03	0.45 - 2.37	0.943	6,524	98
	Model 5	Model 4 + alcohol consumption	0.95	0.38- 2.36	0.906	5,638	88
Black							
	Model 1	Age,Sex	2.16	0.53-8.84	0.283	5,242	66
	Model 2	Model 1 + BMI and hypertension	2.39	0.58-9.82	0.226	5,166	65
	Model 3	Model 2 + LDL-C + HDL-C	N/A				
	Model 4	Model 3 + triglycerides	N/A				
	Model 5	Model 4 + alcohol consumption	N/A				
Mixed							
	Model 1	Age,Sex	N/A				
	Model 2	Model 1 + BMI and hypertension	N/A				
	Model 3	Model 2 + LDL-C + HDL-C	N/A				
	Model 4	Model 3 + triglycerides	N/A				
	Model 5	Model 4 + alcohol consumption	N/A				
Other							
	Model 1	Age,Sex	1.90	1.03- 3.5	0.040	4,608	85
	Model 2	Model 1 + BMI and hypertension	1.91	1.03-3.52	0.039	4,523	84
	Model 3	Model 2 + LDL-C + HDL-C	1.54	0.79-3.00	0.204	4,135	79
	Model 4	Model 3 + triglycerides	N/A				
	Model 5	Model 4 + alcohol consumption	N/A				

Very low Lp(a): Lp(a) <3.8 nmol/. Models with <10 event-per-variable were not reported. BMI: body mass index, CI: confidence interval, HDL-C: high density lipoprotein cholesterol, HR: hazard ratio, LDL-C: low-density lipoprotein cholesterol, N: Number of participants in group, N_{event}: number of events for the group.

Figure S1. Mendelian Randomization methods and sensitivity analyses

Assumption IV1 (the variant is predictive of the exposure) (**A**) can be assessed using the F-statistics when using a two-sample univariable MR-inverse-variance weighted (IVW) analysis. In case the MR-Egger approach is used, weak instrument bias can be assessed using the I^2_{GX} index. Assumption IV2 (**B**; the variant is independent of any confounding factors of the exposure—outcome association) cannot be proven. However, several analyses were undertaken to disprove it or estimate a causal effect in the presence of violation of the assumption. **B1**) Confounding. Confounding is, in theory, limited by the second law of Mendel, which states that genetic variants for different traits are inherited independently. To further limit confounding, the exposure GWAS was corrected using standard methodology and the samples of the exposure and outcome GWAS did not have any overlap. **B2**) Horizontal pleiotropy. MR-PRESSO and leave-one out analyses exclude some, but not as many as median and mode-based analyses, SNPs from the MR estimate using a data-driven or systematic approach. They are especially useful to see whether the results are robust to a few horizontally pleiotropic outliers. Assumption IV3 (**C**; the variant is conditionally independent of the outcome given the exposure and the confounding factors) can also not be proven. We undertook the following steps to detect its violation or to estimate a causal effect in the presence of its violation. First, (**C1**) was explored using the Rücker framework which assesses pleiotropy distribution using heterogeneity in MR-IVW (Cochran Q), MR-Egger (Rücker Q') and by calculating their difference (Q-Q'). The results can be used to move between standard MR methods (MR-IVW fixed and random effects, MR-Egger). Additionally, the I^2 index is calculated to assess potential heterogeneity. **C2**) was assessed using Steiger filtering, which removes variants from the analysis if they are more strongly associated with the outcome than with the exposure. Optionally, it is possible to perform a bi-directional MR to evaluate whether the exposure causes the outcome, or the outcome causes the exposure. This was not performed in the present study.
Finally, weighted median and mode-based analyses were performed as sensitivity analyses which are more robust in terms of violation of assumptions IV2 and IV3. Since these methods allow for half or the majority of the SNPs to be invalid, they have a natural robustness to variants with outlying ratio estimates, and so are not as affected by the presence of a small number of pleiotropic variants as the IVW and MR-Egger methods.

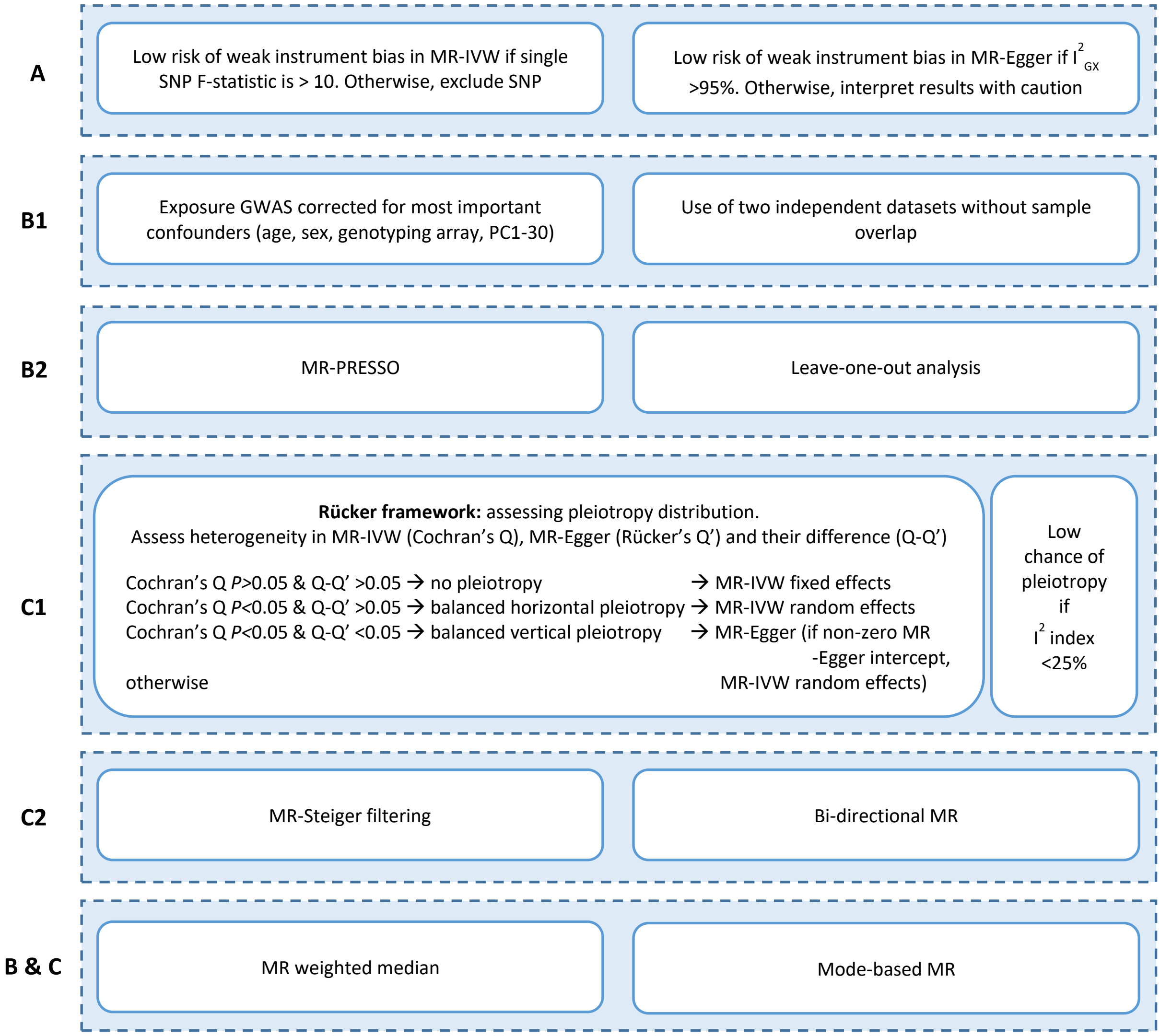

Figure S2. Forest plot illustrating the unadjusted and adjusted hazard ratios of very high Lp(a) values at baseline

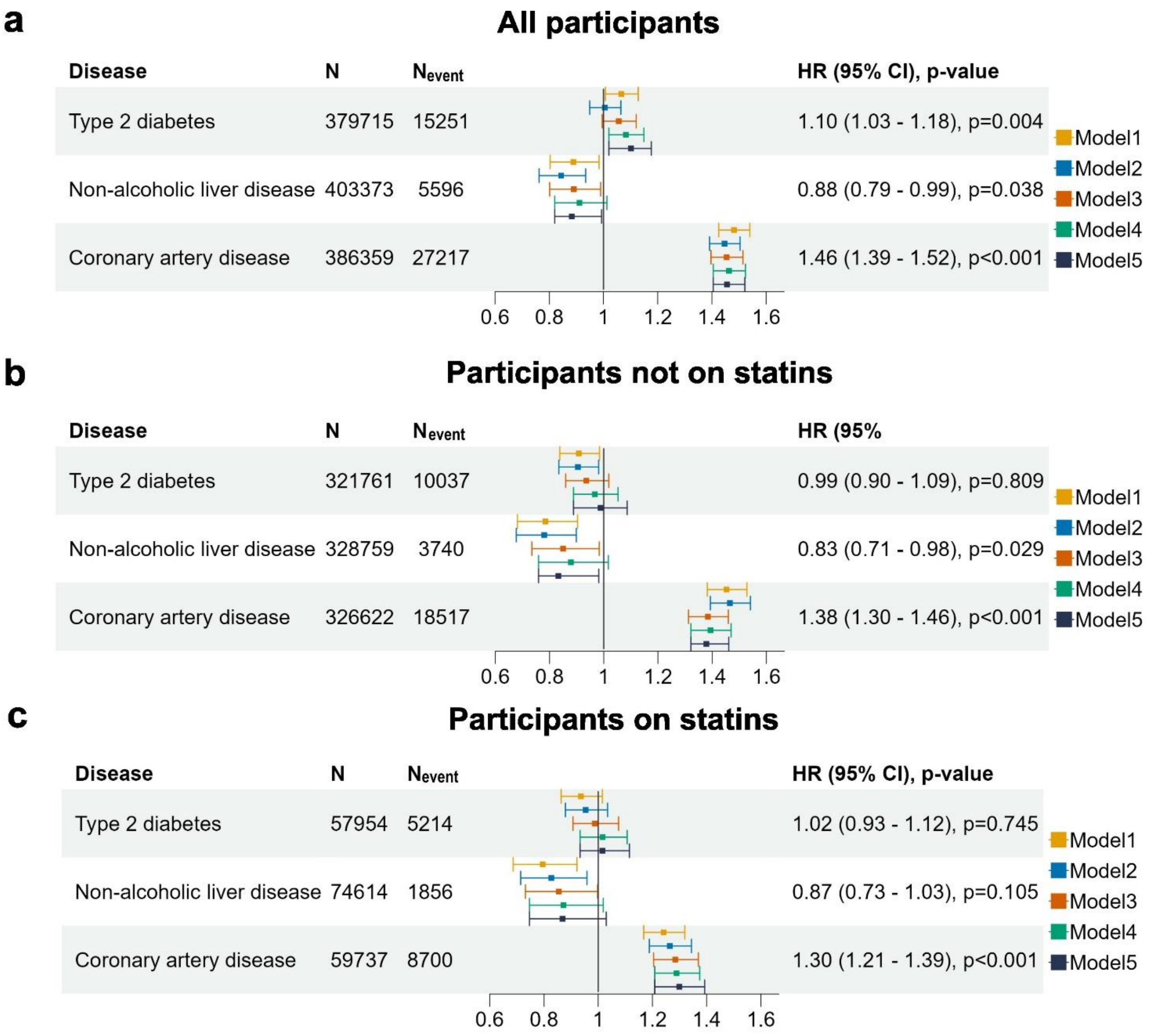

Hazard ratios for diseases in **(a)** all participants, **(b)** participants not using lipid-lowering medications and **(c)** participants using lipid-lowering medications in model 4. Model 1: adjusted for age and sex; Model 2: Model 1 + body mass index and hypertension; Model 3: Model 2 + low-density lipoprotein cholesterol and high-density lipoprotein cholesterol; Model 4: Model 3 + triglycerides; Model 5: Model 4 + alcohol consumption in UK Units (8g pure alcohol) per week at baseline visit. Hazard ratios (HR) with 95% confidence intervals (CI) and p-values of Model 5 are shown for new-onset T2D, NAFLD and CAD. N: Number of

participants, $N_{event:}$ number of events. **Figure S3. Forest plot illustrating the unadjusted and adjusted hazard ratios of inverse rank normalized Lp(a) values at baseline**

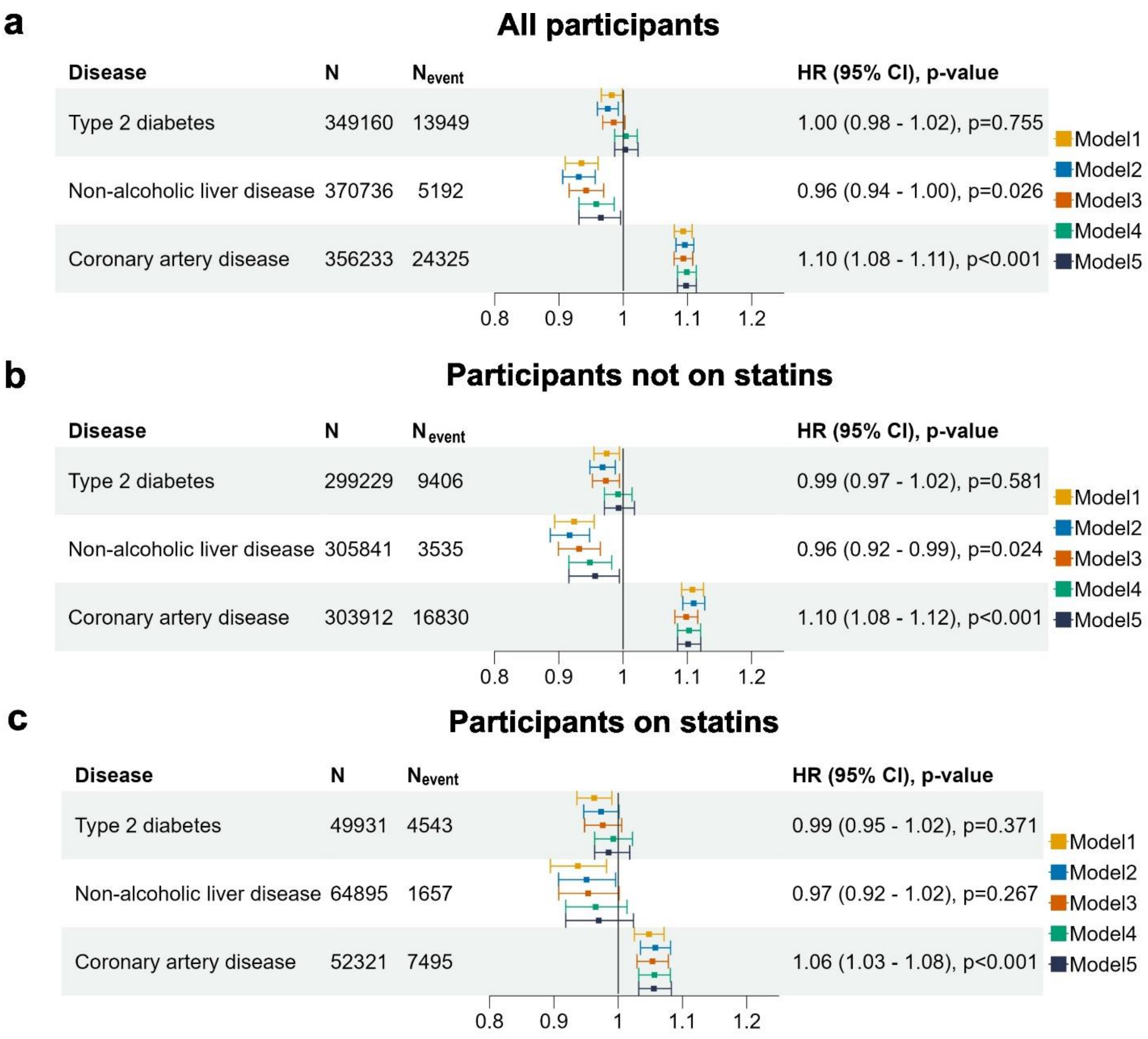

Hazard ratios for diseases in **(a)** all participants, **(b)** participants not using lipid-lowering medications and **(c)** participants using lipid-lowering medications in model 4. Model 1: adjusted for age and sex; Model 2: Model 1 + body mass index and hypertension; Model 3: Model 2 + low-density lipoprotein cholesterol and high-density lipoprotein cholesterol; Model 4: Model 3 + triglycerides; Model 5: Model 4 + alcohol consumption in UK Units (8g pure alcohol) per week at baseline visit.
